# Evaluation of visual pathways using visual evoked potential in patients with diabetic retinopathy

**Published:** 2019

**Authors:** Angela Corduneanu, Veronica Chişca, Natalia Ciobanu, Stanislav Groppa

**Affiliations:** * Department of Ophthalmology, “Nicolae Testemiţanu” State University of Medicine and Pharmacy, Chişinău, Republic of Moldova; ** Department of Neurology, “Nicolae Testemiţanu” State University of Medicine and Pharmacy, Chişinău, Republic of Moldova

**Keywords:** retinopathy, diabetes, visual evoked potential

## Abstract

Visual evoked potential (VEP) is an electrophysiological exploration to detect the response to light stimulus and reveal visual pathways.

**Aim:** VEP study in patients with diabetes mellitus (DM), assessment of cortical and retinal activity, and identifying the role of this investigation in the diagnosis of diabetic retinopathy.

**Methods:** A case-control study conducted to investigate two groups: the first group (G1) included 78 patients (156 eyes) with different stages of diabetic retinopathy, and the second group (G0) included 78 healthy subjects (156 eyes). All subjects have been ophthalmologically and neurologically tested, also using visual evoked potentials. The patients have been exposed to mono-ocular, non-patterned stimuli, using LED-goggles glasses.

**Results:** A serious increase in P100 and N75 wavelength latency in diabetic patients has been observed when compared to healthy subjects (p<0.05), and a N135 value increase in patients with diabetes mellitus (p=0.06). In addition, the amplitude of the P100 wave has changed in diabetic patients in comparison to healthy subjects.

**Conclusions:** Changes in latency of waves registered on the VEP pathway and the amplitude of the P100 wave have been observed in patients with diabetic retinopathy (89.7%), which proved the importance of this study in the diagnosis of diabetic retinopathy and the possibility to examine the prognosis of this disabling disease.

## Introduction

Diabetic retinopathy is the main cause of blindness and visual impairment. It damages the small blood vessels of the retina, leading to progressive vision loss. The link between retinopathy and neuropathy is less investigated [**1**].

As many perceive vasculopathy and neuropathy connected to diabetes, changes along the visual path starting with the retina are to be expected. Visual disabilities in diabetes are a result of vascular disease and metabolic abnormalities that may affect the retina, the optic nerve, and the visual pathways. Metabolic abnormalities of diabetes might cover node cells across the whole retina and macular region. Moreover, neural transmission along the central postural pathways could be delayed [**2**].

Diabetic retinopathy is usually perceived as a retinal vascular pathology, and seldom is seen as a neurosensory disorder, if taken in a broader sense.

The peripheral nervous system disorders caused by diabetes are well investigated, but the changes in the central nervous system and especially their relationship with the visual function are not. Prior to the onset of microvascular lesions, the neuronal retina of diabetic eyes is affected by slight functional changes, which are impossible to detect ophtalmoscopically [**3**]. 

VEP measures how long a visual stimulus needs to get from the eye to the occipital cortex. As the nerve sheath is damaged, the electrical signals need more time, which leads to an abnormal VEP. The prolonged latency of the P100 wave registered in diabetic patients demonstrates that there is a structural damage to the optic nerve fibers. Various pathogenic mechanisms, the origin of which is multifactorial, might be the reason. These might include metabolic and vascular factors, where ischemia and synthesis of advanced glycolysis products can lead to axonal loss caused by diabetes [**4**]. 

## Material and methods 

We conducted a case-control study that included two groups: the G1 study group consisted of 78 patients (156 eyes) with varying degrees of diabetic retinopathy, and the G0 control group included 78 healthy subjects (156 eyes).

We separated the G1 study group into 2 subgroups: 1A group that included patients with proliferative diabetic retinopathy (49 eyes, 32%) and severe form of non-proliferative diabetic retinopathy (41 eyes, 26%), and 1B group included patients with mild non-proliferative retinopathy (29 eyes, 18%) and moderate non-proliferative diabetic retinopathy (37 eyes, 24%). This subgroup separation was performed based on the severity of retinal changes.

The age of patients in the first group G1 varied between 22 and 72 years, and in the second group between 28 and 74 years.

All subjects included in this study were examined ophthalmologically and neurologically, and were also examined through visual evoked potentials. Mono-ocular, non-patterned stimuli, using LED-goggles glasses, were applied.

**Fig. 1:**
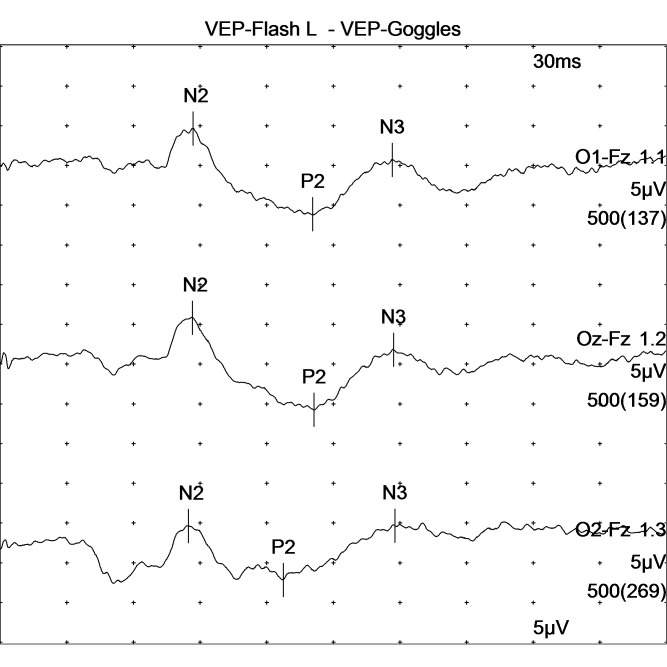
VEP pathway recorded as a result of right eye stimulation in a patient with proliferative diabetic retinopathy shows significantly elevated values of P100 wavelength latency and a decreased amplitude of the P100 wavelength

**Fig. 2:**
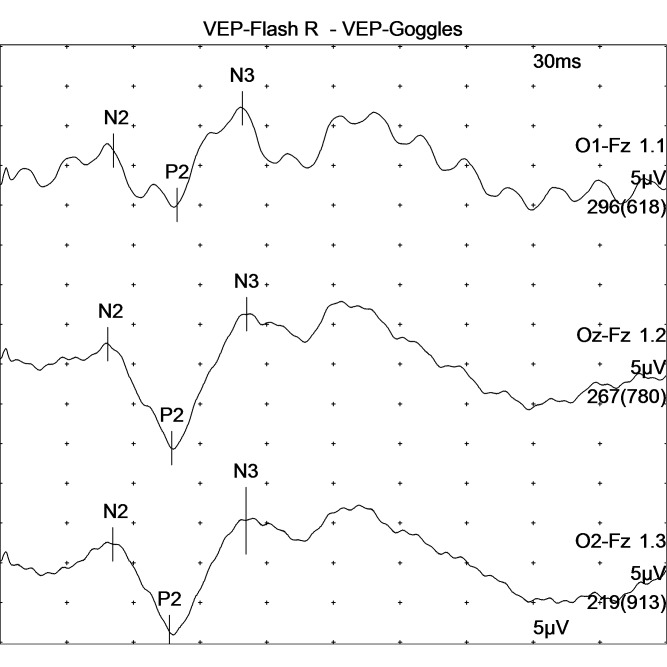
VEP recorded as a result of right eye stimulation in a patient with mild form of non-proliferative diabetic retinopathy shows slightly elevated P100 wavelength and slightly decreased amplitude of P100 wave

## Results

The duration of diabetes in studied patients varied between 2 and 27 years. Type I diabetes was established in 4% of the cases and type II diabetes in 96% of the patients. The gender distribution of patients enrolled in the study was the following: male - 41 patients, female - 37 patients.

Statistically significant elevated values of N75 and P100 waves were found in patients diagnosed with diabetic retinopathy as compared to healthy subjects. At the same time, there was a significant decrease in the amplitude of the P100 wave in patients with diabetes compared to healthy subjects (**Table 1**). Values of P135 latency were insignificantly increased in patients with diabetic retinopathy (p=0,06).

Changes on the PEV pathway were found in 100% of the G1A group and 75% in the G1B group, which represents 89.7% of the study group (G1).

A conclusive increase in P100 wavelength was determined in both groups of diabetic retinopathy (G1B group p=0.001 and G1A p<0.001 group) compared to the control group, whereas the P100 amplitude was significantly reduced only for the group G1A (**Table 2**).

Patients with diabetes showed signs of alterations in optic path integrity, and the presence of prolonged latencies indicated the presence of central neuropathy.

Although there was a significant increase in N75 wavelength latency (p=0,037) and a significant decrease in the magnitude of P100 (p=0,001) in diabetic subjects, no correlation was observed between changes in the VEP pathway and the blood glucose level or duration of diabetes. Similar data was reported in other studies [**5**].

Increased P100 wavelength latency in the group of patients with non-proliferative retinopathy suggested the importance of this method in the early detection of optic lesions in patients with diabetes.

**Table 1:** Average values of VEP waveforms in patients with diabetic retinopathy and in healthy subjects

group	The first group (G1)	The control group (G0)	The p value
latency N75	91,44±18,2	77,46±2,12	<0,001
latency P100	124,52±19,34	99,2±2,65	<0,001
amplitude P100	12,5±11,79	15,31±0,27	0,026
latency N135	158,65±25,06	132,4±3,4	0,06

**Table 2:** The average values of VEP waveforms in the two study subgroups patients

Group	G1A	G1B	t-statistic, p
latency N75	93,5±18,93	84,82±14,09	p=0,0695
latency P100	128,27±21,7	122,09±8,23	p=0,0481
amplitude P100	12,04±6,35	12,68±13,19	p=0,8370
latency N135	158,81±27,12	158,14±17,46	p=0,9192

## Discussion

The visual evoked potential (VEP) is a high sensitive electrophysiological examination to detect changes in visual pathways, especially previous optical lesions. It is also an investigation used to diagnose multiple sclerosis, but unfortunately, there are relatively few studies regarding the importance of this research in patients with diabetes. According to some research data, neuropathy is a significant factor in the pathogenesis of diabetic retinopathy. As neuropathy is a sign of aggravation in diabetes, early clinical symptoms of retinopathy are easy to detect during the electrophysiological tests [**6**]. 

In order to detect demyelination of the optic nerve, the examination of P100 wavelength using VEP is undergone. Demyelinated fibers drive nerve impulses at a lower speed, which explains the increased values of P100 wavelength latency. Hyperglycemia in patients suffering from diabetes trains the polyol pathway, so that glucose is converted to sorbitol and fructose due to aldose reductase enzyme and sorbitol dehydrogenase. The nerve cell is hermetic to some extent; therefore, sorbitol and fructose are in excess. Fructose and sorbitol are active osmotic compounds and by increasing the water content, they generate many other actions, leading to a reduction in Na/ K-ATPase activity, intra-axial sodium accumulation, decreasing the rate of transmission of the nervous impulse. One of the vascular hypotheses indicates the development of ischemia/ hypoxia that leads to the early development of increased vascular resistance with diminished amount of oxygen [**7**-**9**].

Changes in the latency and amplitude of the P100 wavelength in patients with diabetic retinopathy are consistent with source data [**10**,**11**]. Our study has proved that P100 wavelength latency is significantly higher in patients with diabetic retinopathy compared to the control group (p<0,001).

According to our study, changes in the VEP pathway were registered in 100% of the G1A group and 75% in the G1B group, which represent 89,7% of the study group (G1). During the study, we discovered that the P100 wavelength is receding in patients with diabetic retinopathy. Such reduction in P100 amplitude has been registered in other studies too [**2**].

The exact pathophysiology of nerve pathway impairment in diabetic patients hasn't been established yet, but it seems to be multifactorial, including metabolic and vascular factors, and which has obvious similarities with the pathogenesis of peripheral diabetic neuropathy.

Verrotti’s study in 30 patients showed that P100 latency was considerably delayed in patients with diabetes compared to the control group (p<0,01), while latency N75 and P100 amplitude had similar evolution in both groups [**12**].

The Javad Heravian and colleagues study showed an abnormal VEP evolution in 60% of the diabetic patients [**13**]. They also discovered increased P100 wavelengths in patients with diabetic retinopathy but did not detect any relationship between blood glucose levels and P100 wavelength values. The investigations conducted by Li and Yang reported that VEP pathway abnormalities correlate with hyperglycemia [**14**]. But, our study showed no evidence of such a correlation between glycemia and P100 latency in diabetic patients (r=0,12). Chopra D et al. found out that there was a correlation between DM duration and latency of the P100 wave [**15**], but according to our study, there is no significant correlation between the duration of diabetes and the P100 wavelength. 

It is assumed that the prolongation of P100 wavelength in diabetic patients may be the result of structural lesion in the demyelinated fibers of the optic nerve.

We have noticed contradictory research data regarding the VEP changes in patients with diabetes, that is data connected to the percentage and correlations with the duration and the degree of computation of DM. Still, all the reports agree on a definite existence of changes in VEP waves.

The examination of P100 wavelength latency using VEP is therefore a high sensitive method in determining the demyelination of the optic nerve. Demyelinated fibers drive the nerve impulses at a reduced speed, causing a prolonged latency period of the P100 wave, axonal loss, and decreased amplitude of the P100 wave, all of which have lower values in patients with proliferative diabetic retinopathy.

## Conclusions

1. Most patients with diabetic retinopathy attest changes in the VEP tract.

2. The increase of P100 wavelength latency in the group of patients with non-proliferative retinopathy suggests the utility of this method in the early detection of optic lesions at diabetic patients.

3. Early diagnosis of central neuropathy can provide an early opportunity for proper management.
